# An assessment on potential risk pathways for the incursion of highly pathogenic avian influenza virus in backyard poultry farm in Bangladesh

**DOI:** 10.14202/vetworld.2020.2104-2111

**Published:** 2020-10-09

**Authors:** Kamrul Islam, Md. Murshidul Ahsan, Shovon Chakma, Kinley Penjor, Mukti Barua, Mohammad Shah Jalal, Abdullah Al Momen Sabuj, Zakia Tabassum Ani, Abdul Ahad

**Affiliations:** 1 Institute of Epidemiology, Disease Control and Research, Mohakhali-1212, Dhaka, Bangladesh; 2 Department of Chemical and Biomolecular Engineering, National University of Singapore, 4 Engineering Drive 4, Singapore 117585; 3 Bhutan Agriculture and Food Regulatory Authority, Bhutan; 4 Department of Animal Science and Nutrition, Faculty of Veterinary Medicine, Chattogram Veterinary and Animal Sciences University, Chattogram, Bangladesh; 5 Department of Microbiology and Veterinary Public Health, Faculty of Veterinary Medicine, Chattogram Veterinary and Animal Sciences University, Chattogram, Bangladesh; 6 Department of Microbiology and Hygiene, Bangladesh Agricultural University, Mymensingh-2202, Bangladesh; 7 Department of Sociology, Faculty of social sciences, University of Chittagong, Bangladesh

**Keywords:** control options, highly pathogenic avian influenza, live bird market, prevention, risk assessment, risk pathways

## Abstract

**Background and Aim:**

Highly pathogenic avian influenza (HPAI) is a deadly virus of zoonotic potential. The study mainly aims to determine the risk pathways (RPs) for the probable incursion of HPAI virus (HPAIV) in backyard poultry in Bangladesh.

**Materials and Methods:**

The study involves expert elicitation technique. The concept map determines the possible RPs. The map consists of 16 concepts, each with nodes from which probabilities of an event originates. These probabilities are described by qualitative descriptors ranging from negligible to high. Risk assessment has been performed using the subjective risk assessment tool.

**Results:**

The tool demonstrates positive correlation among groups of experts in the level of agreement by scoring RP; however, the level of agreement varies from 71% to 93% among group of experts. The median risk score of viral incursion through the “Exposure of backyard poultry with farm poultry in the trading market” was 11 and ranked as top, followed by “Contaminated live bird market environment” and “Sharing common scavenging space with migratory birds” (median risk score, 10.5; rank, 2), and “Scavenging of infected slaughtered poultry remnants by backyard poultry” (median risk score, 5.3; rank, 3) when no control options were applied along with the RPs. After applying or considering control option along with contaminated live bird market environment, the median risk score was reduced to 5.0. Applying a specific control option along with each RP reduced estimated median risk scores for HPAIV incursions.

**Conclusion:**

This study provides an insight into the incursion risks of HPAIV through various RPs in backyard poultry in Bangladesh.

## Introduction

Over the past decade, numerous highly pathogenic avian influenza virus (HPAIV) outbreaks in poultry holdings were recorded in South Asian countries. The region is considered as an epicenter for the emergence of infectious diseases with pandemic potential such as the goose/Guangdong lineage of HPAIV H5Nx [1]. This is due to complex ecological, biological, social-economic, and technological processes interlinked in ways that enable microorganisms to exploit new ecological niches [2]. HPAI H5N1 virus outbreaks were first announced by the Government of Bangladesh (GoB) in March 2007 in commercial poultry farms [3] and were recognized in humans for the 1^st^ time in May 2018 [4]. A total of eight H5N1 human cases were reported so far, including a fatality [4]. In Bangladesh, HPAIV H5N1 infections have coexisted with low pathogenic avian influenza infections, predominantly of the H9N2 subtype. So far, three clades (2.2.2, 2.3.4.2, and 2.3.2.1a) of H5N1 have been detected in Bangladesh [5-7].

Bangladesh is a nation of about 150 million individuals, with 64% residing in rural settlements [8]. Around 71% of the rural community households raise non-commercial backyard poultry [9] with close contacts between poultry and people [10]. In the rural community household in Bangladesh, poultry farmers get into very close contact with backyard poultry consistently in their everyday life, for example, while (1) placing them into sheds, (2) feeding sick chicken by hand, and (3) slaughtering sick or healthy poultry [11]. Although the major source of HPAIV introduction into poultry sectors (backyard and commercial) in Bangladesh is believed to be from waterfowls (ducks) through direct or indirect contacts with migratory wild birds [12-14], the pathway of its introduction and subsequent spread in backyard poultry has not been established completely. A matched case-control study was carried out in backyard poultry farms in 2007 to evaluate the risk factors for transmission of HPAIV infections in backyard poultry in Bangladesh. The results showed that (1) offering slaughter remnants of purchased chickens to backyard chickens, (2) presence of water bodies in close proximity, and (3) contact with pigeons were the independents risk factors for infection with HPAIV H5N1 [15]. Although the GoB and other collaborating international organizations allocate resources for rapid detection, control, and surveillance system of HPAI in humans and commercial poultry farms, limited attention had been paid to backyard poultry. Since 2007, more than 550 outbreaks have been reported in Bangladesh, of which nearly 60 outbreaks originated in backyard holdings [5,15-16].

Expert consultation is an alternative and valuable way of collecting knowledge in a field where accurate and unbiased field data are not available [17,18]. Information on the introduction and spread of HPAI (i.e., risk pathways [RPs]) among backyard poultry operations in Bangladesh is sporadic. Information from neighbor countries in South and Southeast Asia are not transferrable to Bangladesh because of the differences in weather patterns, conditions of wild bird species and migratory bird patterns, backyard poultry raiser’s biosecurity practices, etc., which result in variation in pathways for the introduction of the virus in backyard poultry in Bangladesh. The existing hypothesis about the maintenance of the HPAIV infection among the poultry population in Bangladesh is that there is a continuous circulation of the HPAIV takes place involving all poultry sectors (both backyard and commercial) as well as the market value chains in an “infection cycle.” The role of backyard poultry farms is considered to be very important in maintaining the circulation of HPAIV due to a lack of biosecurity. The RPs for incursion of the HPAIV infection in the backyard poultry farm in Bangladesh are, however, not yet well understood and documented.

Therefore, the study was aimed to determine the RPs for incursion of HPAIV in backyard poultry in Bangladesh. 

## Materials and Methods

### Ethical approval and informed consent

No ethical approval was required; however, verbal consent was taken from the experts.

### Identification of experts

Eight veterinarians from the government, non-governmental organizations, and international multilateral organizations were selected based on their expertise in the respective field. Those with high-level authority and/or special knowledge were selected for the purpose and were contacted over the phone. The study aim and method were explained to them and they were asked for their consent to participate in this study. Subsequently, the questionnaire and instructions were provided to participants who were requested to respond within 4 weeks. The experts were randomly allocated to four groups, comprising two members in each group. Each group was designated by the capital letter A, B, C, and D.

### Study design

This qualitative expert elicitation exercise consisted of three stages. At the first stage, selected experts were invited to participate in a video conference arranged in February 2017. The first video conference was executed to introduce experts to each other as a group and to identify RPs for a possible introduction of the HPAIV in backyard poultry. The term “backyard poultry” was used to refer free-range chickens, ducks and/or geese, and pigeons, including juveniles and adults with an average flock size of 10. The term “scavenging” was used to refer to the chicken behavior of “roaming in places in search of food, scratching, and eating food from those places.”

**Figure-1:**
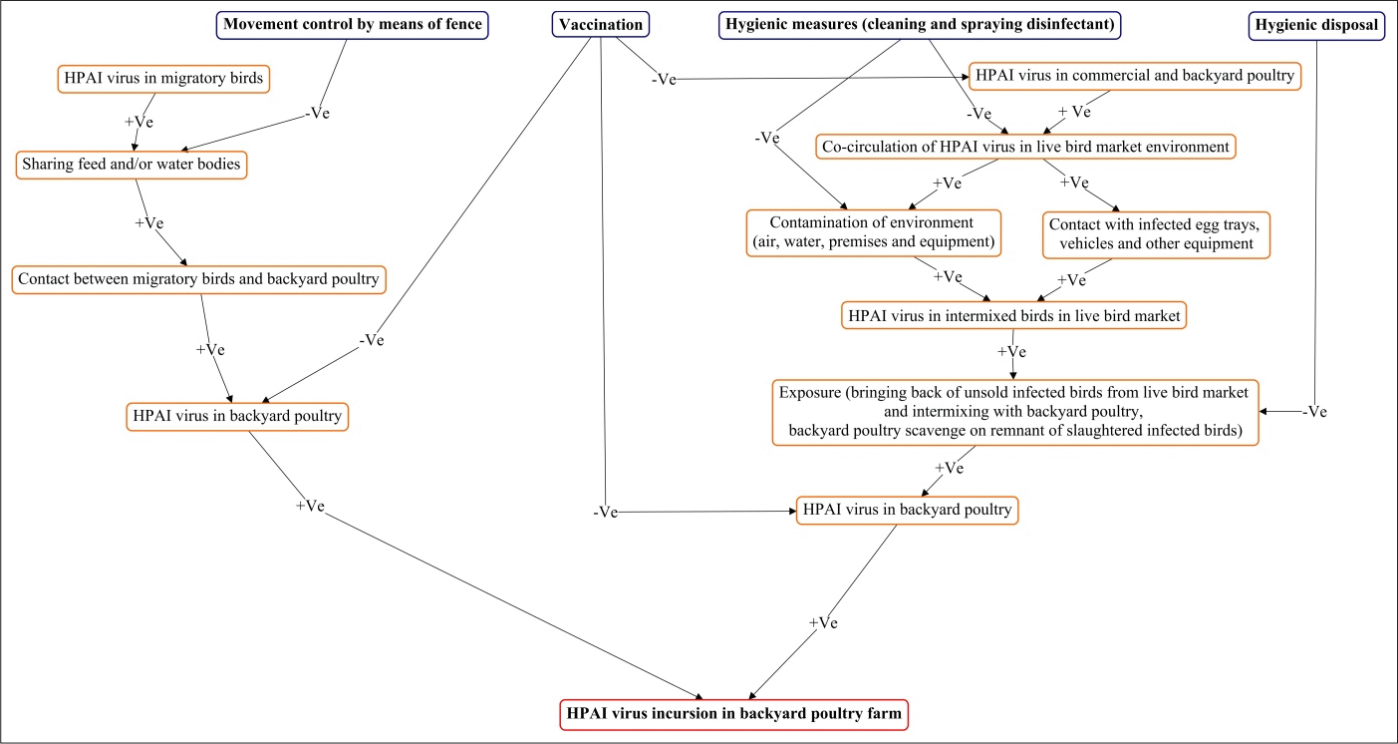
Concept map of risk pathways for probable incursion of highly pathogenic avian influenza virus in backyard poultry farm.

A concept map (Cmap)/cognitive map (Figure-1) was generated following this conference. Subsequently, at the second stage, experts were requested to complete the questionnaire that was sent through email, to conduct likelihood/probability estimates on various RPs. In the third stage, a video conference was executed in April 2017 among groups of experts where scores of the initial estimates were presented and discussion among experts was encouraged. Experts were asked as a group to estimate likelihood/probability and consequences associated with each RP. A numeric score ranging from 1: Negligible to 5: Catastrophic was assigned as a consequence score for each RP. Similarly, a score ranging from 1: Negligible to 5: High was assigned as likelihood/probability score for each of the RPs. The qualitative descriptors are defined in Tables-1 and 2. Each expert must assign numerical values (score) for likelihood and consequences. These can be single numbers (for example 4), ranges (e.g., 3-5), or ranges with mid-point (e.g., 4-6-9). Multiplying likelihood and consequence score provided the final estimate or risk scores corresponding to each RP (hazard). Subsequently, RPs were ranked based on the median value of the midpoint risk score. Rank 1 corresponds to the highest risk. The results were compiled into a risk matrix using subjective risk assessment (SRA), a Flash-based tool [19]. Expert groups provided scores along with each RP before/without and after/with considering disease control options. The method was based on a similar structure described by Carey and Burgman [20].

**Table 1:** Likelihood descriptors in the risk assessment.

Score	Qualitative descriptors	Description
1	Negligible	The event may occur in exceptional circumstances
2	Low	The event is unlikely to occur
3	Slight	The event is likely to occur with a low probability
4	Moderate	The event is likely to occur with a high probability
5	High	The event is anticipated to occur in most circumstances

**Table 2:** Consequences descriptors in the risk assessment.

Score	Qualitative descriptors	Description
1	Negligible	Public health consequences, effect on poultry farming and trading, impact on production and consumption of poultry meat and eggs, and financial loss due to the incursion of HPAI are negligible
2	Low	Public health impact, consequences on poultry farming and trading, impact on production and consumption of poultry meat and eggs, and financial loss due to the incursion of HPAI are low
3	Moderate	Public health consequences, effect on poultry farming and trading, impact on production and consumption of poultry meat and eggs, and financial loss due to the incursion of HPAI are moderate
4	High	Public health costs, consequences on poultry farming and trading, effect on production and consumption of poultry meat and eggs, and financial loss due to the incursion of HPAI are high
5	Catastrophic	Public health impact, effect on poultry farming and trading, consequences on production and consumption of poultry meat and eggs, and financial loss due to the incursion of HPAI are catastrophic

HPAI=Highly pathogenic avian influenza

### Terminology

Qualitative descriptors were used as adjectives to determine the likelihood and consequences associated with RPs (Tables-1 and 2). To reduce the possibility of bias, expert groups discussed, reviewed, and finally adopted descriptors with few modifications from Corbellini *et al*. [21]. Four hypothetical RPs have been determined based on expert discussion (Figure-1). These are as follows:

a. RP-1: Sharing common scavenging space with migratory birds
b. RP-2: Exposure of backyard poultry with farm poultry (backyard and commercial poultry holdings) in the trading market
c. RP-3: Contaminated live bird market (LBM) environment
d. RP-4: Scavenging of infected slaughtered poultry remnant by backyard poultry.

Four hypothetical control options were considered along with each RP (Figure-1) based on the literature available and expert discussion. These are as follows:

a. Movement control by means of fencing off backyard poultry along RP-1
b. Vaccination along RP-2
c. Hygienic measures (cleaning and spraying disinfectant) along RP-3
d. Hygienic disposal of slaughtered poultry remnant or waste along RP-4.

### Cmap scenario

As described in the study design, a Cmap/cognitive map using Cmap Tools, version 6.04 (https://cmap.ihmc.us/) (Figure-1) was constructed to determine the possible RPs for the incursion of the HPAIV in backyard poultry. The map included 16 concepts (Figure-1). The map consisted of nodes with concepts from which the likelihood/probabilities of an event originated (Figure-1). These likelihood/probabilities were described by qualitative descriptors ranging from negligible to high (Tables-1 and 2). The concepts included different RPs for the probable incursion of HPAIV in backyard poultry and control options. The association between cause and impact was demonstrated by elicitation. The proposition was constructed using more than 2 concepts. Symbols (+Ve or –Ve) were used to reflect the relationship among concepts. Positive symbol (+Ve) indicates a positive association between concepts. Negative symbol (−Ve) indicates a negative association between concepts.

### Risk matrix

A risk assessment was conducted, and results were compiled into a risk matrix using the SRA tool [19]. The analysis was conducted to evaluate the RPs for a probable incursion of HPAIV in backyard poultry. The effectiveness of different control options along with each RP was analyzed. The data were generated by scoring each pathway with and without control options. To avoid bias, numbers were used to value the descriptors. The function of likelihood/probabilities and consequences was used to estimate the risk. Earlier described likelihood/probabilities and consequences were used in the risk assessment with minor modifications [21]. A table was generated during analysis (Table-3) based on a score related to the qualitative descriptors.

**Table 3:** Combination of likelihood and consequences to estimate the risk of each pathways without/no and with applying control option.

Likelihood		Consequences				

		Negligible	Low	Moderate	High	Catastrophic
				
		(1)	(2)	(3)	(4)	(5)
High	(5)	5	10	15	20	25
Moderate	(4)	4	8	12	16	20
Slight	(3)	3	6	9	12	15
Low	(2)	2	4	6	8	10
Negligible	(1)	1	2	3	4	5

## Results and Discussion

### Participants for the expert elicitation

All eight selected experts completed and returned the questionnaire electronically. Of the eight participants, three were governmental employees, two were affiliated with a private poultry company, and one each was employed in academia, an international agency and a non-government agency with a mean of 9.5 years of professional experience in the livestock and poultry sector.

### Construction of Cmap and identification of RPs

Following discussion among groups of experts, a Cmap (Figure-1) was constructed to determine possible RPs. Migratory or aquatic birds are known reservoirs of avian influenza virus [16]. Each year thousands of wild birds migrate to Bangladesh from countries as far as Siberia for wintering purposes [21]. They frequently share food sources and water bodies with backyard poultry and provoke chances for HPAIV introduction into a new susceptible host through direct or indirect contacts. Movement restriction of backyard poultry by means of fencing off may reduce contact with migratory birds. For that, movement control was considered a control option. Second, birds are brought to LBMs from commercial and backyard poultry farms sourced in different regions of Bangladesh. If these commercial and backyard poultry harbor the HPAIV, they may contaminate the LBM environment and infect other healthy birds in the market and spread the HPAIV to backyard poultry, particularly when village people purchase live birds from local LBM and introduce them to their backyard flock. In some instances, village people bring back their unsold birds from the LBM to their backyard flock. Therefore, exposure of backyard poultry in the LBM and exposure to contaminated LBM environment were considered two other RPs. Vaccination of poultry in farms may reduce HPAIV shedding and circulation among birds [22]. Hence, vaccination was considered as a control option in backyard poultry. In LBM, proper hygienic measures (cleaning and spraying disinfectant) could be an effective control option. Finally, village people purchase poultry from LBM and slaughter it at home. Backyard poultry scavenges on remnants of those slaughtered poultries. This exposure might be a risk for the incursion of HPAIV in backyard poultry of a backyard farm. For this RP, hygienic disposal of the remnant of slaughtered poultry was considered as an effective control option.

### Evaluation of likelihood of HPAI incursion in backyard poultry

The likelihood of incursion of HPAIV through various RPs was analyzed by the SRA tool. The result showed an overall positive correlation between groups of experts on the degree of agreement on scoring RPs (Table-4). Four hypothetical RPs were determined based on Cmap and presented to experts. Experts were requested to give likelihood and consequence scores based on above described scenarios before and after applying control options along with each RP.

**Table 4:** Level of agreement among groups on scoring and ranking of RPs.

Pair No.	Groups of experts	Agreement (%)
1	A-C	93
2	B-D	85
3	A-B	82
4	C-D	79
5	B-C	79
6	A-D	71

RP=Risk pathway

### The level of agreement among groups on scoring and ranking RPs

The level of agreement varies among groups from 71% to 93%. The level of agreement was higher between Group-A and Group-C (93%), whereas the low-level similarity was found between Group-A and Group-D (71%) in the agreement of scoring and ranking RPs for the possible incursion of HPAIV (Table-4).

### Ranking of RPs

**Figure-2:**
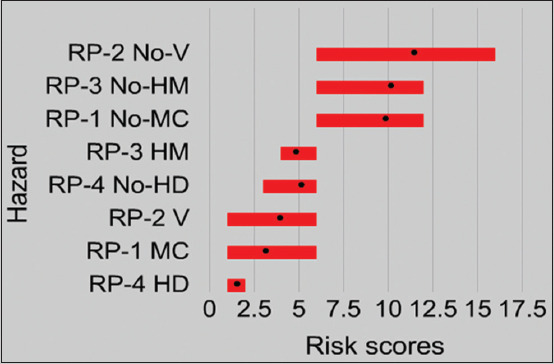
Risk plot showing ranking of risk pathways with and without control options. (In the risk plot, black dots on bars represent averages of expert group’s midpoint risk scores. RP, risk pathway; No-V, no vaccination; V, vaccination; No-HM, no hygienic measures; HM-hygienic measure; no-MC, no-movement control, MC, movement control; No-HD, no hygienic disposal; HD, hygienic disposal; hazard, risk pathways).

The distribution of median risk scores for each RP with and without applying control options is reported in Tables-5 and 6, respectively, and plotted in Figure-2.

**Table 5:** Scoring and ranking of RPs before/without applying control options.

RPs	Definition	Median risk score	Rank
RP-2	Exposure of backyard poultry with farm poultry (backyard and commercial poultry holdings) in the trading market	11.0	1
RP-3	Contaminated LBM environment	10.5	2
RP-1	Sharing common scavenging space with migratory birds	10.5	2
RP-4	Scavenging of infected slaughtered poultry remnant by backyard poultry	5.30	3

LBM=Live bird market, RP=Risk pathway

**Table 6:** Scoring and ranking of RPs after/with applying control options.

RPs	Definition	Control options	Median risk score	Rank
RP-3	Contaminated LBM environment	Hygienic measures (cleaning and spraying disinfectant)	5.0	1
RP-2	Exposure of backyard poultry with farm poultry (backyard and commercial poultry holdings) in the trading market	Vaccination	3.8	2
RP-1	Sharing common scavenging space with migratory birds	Movement control by means of fencing off backyard poultry	3.0	3
RP-4	Scavenging of infected slaughtered poultry remnant by backyard poultry	Hygienic disposal of slaughtered poultry remnant	1.5	4

LBM=Live bird market, RP=Risk pathway

Looking at scenario “a” (no control options), exposure of backyard poultry with other poultry (backyard and commercial poultry holdings) in the trading market is top ranked (RP-2; median risk score, 11; rank 1) followed by contaminated LBM environment (RP-3; median risk score, 10.5; rank, 2), sharing common scavenging space with migratory birds (RP-1; median risk score, 10.5; rank, 2), and scavenging of infected slaughtered poultry remnant by backyard poultry (RP-4; median risk score, 5.3; rank, 3) (Table-5). Scenario “b” (control options are applied) reduces the risk of viral incursion along with each RP. The risk score and rank of RPs are as follows: Exposure to contaminated LBM environment (RP-3; median risk score, 5; rank, 1) followed by exposure of backyard poultry with other poultry (commercial and backyard poultry farm) in the LBM (RP-2; median risk score, 3.8; rank, 2), sharing common scavenging space with migratory birds (RP-1; median risk score, 3; rank 3), and scavenging of infected slaughtered poultry remnant by backyard poultry (RP-4; median risk score, 1.5; rank, 4) (Table-6).

Given vaccination as a control option along with exposure of backyard poultry with farm poultry (backyard and commercial poultry holdings) in the trading market (RP-2), the median risk scores reduced to around one-third of the initial median risk score (from 11 to 3.8). The low prevalence of HPAIV strains in backyard poultry and on commercial farms compared to LBMs suggested that keeping birds in confined areas play a key role in the control of HPAIV [23]. Vaccination or immunization as a control option against specific HPAI strain in poultry farms may reduce shedding and circulation of HPAIV through triggering host immunity, which then lessens the viral load in the LBM environment [22]. This, in turn, may disconnect viral transmission in backyard or commercial poultry, further lessening the viral load in the LBM, blocking exposure due to bringing back unsold birds from LBM intermixed with backyard poultry, and finally disrupting the incursion of HPAIV to backyard poultry farms (Figure-1).

The LBM is the central point of poultry trading and a common interface between poultry and humans. Hygienic measures (cleaning and spraying disinfectant) at LBMs reduced the median risk score from 10.5 to 5. This finding is supported by a meta-analysis conducted by Zhou *et al*. [24], who found that the risk of acquiring AI infection was significantly lower in LBMs that conduct cleaning and disinfection (odds ratio = 0.35; 95% confidence interval, 0.17-0.73) compared with those that did not. In Bangladesh, most of the poultry vendors in the LBM keep their poultry stalls adjacent to one another, thereby facilitating HPAIV transmission between stalls [25]. Infected poultry from LBM may transmit the virus to backyard poultry likely through intermixing of unsold and relocated poultry with backyard poultry. Reversely, local vendors trade backyard birds and eggs of local poultry to local rural LBM or urban LBMs. During this trading bamboo baskets, vehicles and egg trays may act as a source to contaminate LBM environments [3,15,26]. The Food and Agricultural Organization of the United Nations (FAO) conducted a sink surveillance study in the LBMs of Dhaka, Bangladesh. High prevalence of avian influenza (85%) has been found in LBMs in Dhaka where H5 was the most prevailing subtype reaching up to 80% frequency of detection as of April 2016 [27]. Marinova-Petkova *et al*. [5] detected 30 HPAIV H5N1 isolates in Dhaka city LBMs. The findings support the potential role of LBM in circulating and maintaining HPAIV H5N1 subtype in Bangladesh [28-30]. FAO and United States Agency for International Development and circumstantial evidence suggest that better biosecurity is required to intercept HPAIV transmission and circulation in the LBM [7,31,32].

Fencing off backyard poultry reduced the median risk score from 10.5 to 3 (Tables 5 and 6). Migratory birds are considered as a potential carrier of avian influenza virus [16,33]. A number of HPAI outbreaks were reported in backyard poultry in China, Korea, and Japan during 2010-2015 [34-36], Europe in 2014-2015 [37], and North America in 2014-2015 which were considered linked with waterfowl migration [38,39]. Migratory birds typically share food sources and water bodies with backyard poultry which increases the chance of HPAIV. Thus, migration and subsequent intermixing of migratory birds with backyard poultry could play an important role in the incursion of HPAIV [37]. Movement restriction by means of protective fencing made of cheap materials (i.e., bamboo) may reduce possible exposure, decreasing the chance of HPAI introduction in backyard poultry [15,16,40].

Hygienic disposal of slaughtered poultry remnants led to a decline of the median risk score from 5.3 to 1.5. Stalls with live poultry are found in every village market throughout the country. Village people sell poultry to local vendors (known as “forea”) at the local LBM who buy poultry in bulk from the local backyard and commercial poultry farms. The forea sells poultry to larger LBMs in the city. Due to fear of financial loss, sometimes even clinically diseased poultry is sold. Diseased poultry is cheaper which encourages other villagers to buy these birds preferentially. They purchase live poultry and slaughter them at home. Slaughtered remnants and inedible portions from poultry are usually disposed of in an unsafe way or a fed to backyard poultry. This practice enhances incursion risks grossly [15,16,34,41]. Therefore, hygienic disposal of slaughtered poultry remnants should be recommended. The main limitation of this study is uncertainty and bias. These uncertainties arise from, for example, inconsistent interpretation of words or imprecise terminologies, language barriers, and vagueness.

## Conclusion

This elicitation of expert’s opinion highlights useful information about possible RPs through which introduction of HPAIV could occur in backyard poultry in Bangladesh. The various estimates generated (by expert groups) in this study could be used as input values in a risk assessment to inform biosecurity practices to mitigate the risks and will also help in validating some of those estimates. This study demonstrates possible RPs through which HPAIV H5N1 could be entered, and effective control measures are required to employ which could minimize the risk of HPAIV incursion in backyard poultry in Bangladesh.

## Authors’ Contributions

KI, SC, and KP designed and carried out the study. SC, KI, MB, and KP contributed in data collection, analysis, and writing this article with the support from MMA, ZTA, MSJ, AAMS and AA. MMA, ZTA, and AA revised the drafted article and given feedback on the contents. The final version of the manuscript was reviewed and approved by all authors.
